# Interments in the City and Liberties of Philadelphia, from the 9th of October to the 16th

**Published:** 1841-10-23

**Authors:** 


### HEALTH OF THE CITY.

Interments *in the City and Liberties of Philadelphia, from the 9th of October to the 16th.*

| Diseases. | Adults. | Children. | Diseases. | Adults. | Children. |
|---|---|---|---|---|---|
| Apoplexy | 1 | 0 | *Brought forward,* | 32 | 45 |
| Cancer, | 1 | 0 | Inanition, | 0 | 1 |
| Casualties, | 0 | 1 | Jaundice, | 1 | 0 |
| Croup, | 0 | 6 | Lyryngeal Phthisis, | 0 | 1 |
| Consumption of the lungs, | 11 | 2 | Marasmus, | 0 | 2 |
| Convulsions, | 0 | 3 | Neglect, | 0 | 1 |
| Diarrhœa, | 1 | 1 | Old age, | 2 | 0 |
| Dropsy of the head, | 0 | 5 | Purpura, | 0 | 1 |
| —— Heart, | 1 | 0 | Scirrhus, | 2 | 0 |
| Disease of the Brain, | 0 | 1 | Scrofula, | 1 | 0 |
| —— Heart, | 1 | 0 | Softening of Brain, | 1 | 0 |
| —— Lungs, | 0 | 1 | Small pox, | 1 | 2 |
| —— Liver, | 1 | 0 | Still-born, | 0 | 4 |
| —— Spine, | 0 | 1 | Suicide, | 1 | 0 |
| Drowned, | 0 | 1 | Teething, | 0 | 1 |
| Dysentery, | 1 | 0 | Unknown, | 2 | 0 |
| Debility | 0 | 9 | | — | — |
| Erysipelas, | 0 | 1 | Total, 102— | 44 | 58 |
| Fever, | 2 | 0 | | | |
| —— Congestive, | 2 | 0 | | | |
| —— Intermittent, | 1 | 0 | | | |
| —— Remittent, | 3 | 0 | | | |
| —— Bilious, | 1 | 2 | | | |
| —— Nervous, | 0 | 1 | | | |
| —— Typhus, | 1 | 1 | | | |
| —— Typhoid, | 1 | 1 | | | |
| —— Scarlet, | 0 | 1 | | | |
| Gangrene of Lungs, | 0 | 1 | | | |
| Inflammation of the Brain, | 0 | 2 | | | |
| —— Bronchi, | 1 | 2 | | | |
| —— Breast, | 0 | 1 | | | |
| —— Stomach, | 0 | 1 | | | |
| —— Liver, | 1 | 0 | | | |
| —— Uterus | 1 | 0 | | | |
| | — | — | | | |
| *Carried forward,* | 32 | 45 | | | |

Of the above, there were under 1 year, 18

| | |
|---|---|
| From 1 to 2 | 18 |
| 2 to 5 | 12 |
| 5 to 10 | 6 |
| 10 to 15 | 2 |
| 15 to 20 | 3 |
| 20 to 30 | 6 |
| 30 to 40 | 11 |
| 40 to 50 | 7 |
| 50 to 60 | 6 |
| 60 to 70 | 8 |
| 70 to 80 | 3 |
| 80 to 90 | 2 |
| 90 to 100 | 1 |
| Total, | 102 |

Of the above there were 4 from the almshouse, 8 people of colour, and 1 from the country, which are included in the total amount.

